# Incidence of Plasmodium infections and determinant factors among febrile children in a district of Northwest Ethiopia; a cross-sectional study

**DOI:** 10.1186/s40794-018-0069-1

**Published:** 2018-08-10

**Authors:** Tadesse Hailu, Megbaru Alemu, Wondemagegn Mulu, Bayeh Abera

**Affiliations:** Department of Medical Microbiology, Immunology and Parasitology, Bahir Dar University, PO Box 79, Bahir Dar, Ethiopia

**Keywords:** Plasmodium, Incidence, Bed net, Sleeping time, Stagnant water

## Abstract

**Background:**

According to the Ethiopian national malaria indicator survey of 2015, the highest burden of Plasmodia infection resided among the school-age children. Even though several studies revealed various determinant factors of childhood malaria, consistent findings are not reported across the nation and elsewhere. This in turn creates obstacles in implementing exactprevention and control measures in the study area in particular and the country at large.

**Objectives:**

The aim of this study is to determine the incidence of Plasmodium and determinant factors among febrile children in Northwest Ethiopia.

**Methods:**

This cross-sectional study was conducted from April–August 2016. Blood samples were collected from febrile children selected by systematic random sampling. Thin and thick blood films were prepared and stained with Giemsa. Statistical analysis was done via SPSS version 20 statistical software and data were summarized with percentages and frequencies. The bi-variate and multi-variate logistic regressions were used to measure strength of association between Plasmodium infection and determinant factors, and to rule out confounders, respectively.

**Result:**

Among a total of 333 febrile children, 146 (43.8%) were positive for the Plasmodia. The prevalence of plasmodium infection was 47%, 50%, and 40%, among the age groups of 6–8, 9–10 and 11–14 years, respectively. Prevalence of plasmodium among male and female children was 44.2% and 43.5%, respectively. Shorter distance from stagnant water (AOR (adjusted odds ratio) =43, 95% CI (confidence interval):2.8–680.7; *P* < 0.01), family size (AOR =14.7, 95% CI:(1.4–151.2; *P* = 0.02), outdoor sleeping (AOR =36.6, 95% CI:2.4–554.2; *P* < 0.01, irregular bed net use (AOR =21.1, 95% CI:2.9–154.7; *P* < 0.01), and late bed time (AOR =31.9, 95% CI:2.8–371.3; *P* < 0.01) showed statistically significant association with plasmodium infection.

**Conclusion:**

The incidence of Plasmodium infection is high among febrile children in the study area. Shorter distance from stagnant water, larger family size, outdoor sleeping, irregular bed net use, and late night sleeping are the major determinant factors for the high incidence of malaria. Therefore, community mobilization and health education should focus on the specific determinant factors of plasmodium infection to alleviate incidence of malaria among the school children.

## Background

Malaria is one of the world’s greatest challenges to be considered especially in resource poor countries of the tropics and subtropics [1]. In 2016, there were approximately 216 million new malaria cases and 445, 000 malaria deaths worldwide and the African Region harbored 88% of new malaria cases and 90% of malaria deaths [2]. Of these mortalities, 60% occur in the sub Saharan African children [3].

Malaria is also the leading public health problem and cause of outpatient visit accounting for 12% of cases in Ethiopia [4]. It is estimated that about 65% of the population are at risk of acquiring malaria at any one time [5]. *Plasmodium falciparum* represents about 65–75% of the total reported malaria cases, relative frequency varies in time and space within given geographical ranges [2].

Previous studies revealed that the prevalence of malaria is still high in different endemic areas of the country. For instance, high incidence of malaria among febrile individuals was reported in different parts of northwest Ethiopia including Pawe (51.5%) [6], Dembecha (52%) and Quarit (72.7%) [7]. Despite the remarkable reduction in the malaria mortality in children under 5 years in Ethiopia, the school-age children are yet not addressed in terms of prevention and control of malaria in the nation. This could be in part due to the fact that, the school-age children sleep under nets less often than any other age group in many areas of Africa. In such areas there exists a propensity for mothers sleeping under nets with their young children, while older children slept under nets less frequently [8]. Surveys conducted in several African nations revealed that school-aged children were the group least likely to sleep under an ITN which left more than 30% of school-aged children unprotected [9].

Previous studies revealed different determinant factors of childhood malaria in malaria endemic areas such as knowledge of malaria transmission [10], bed net utilization [11], sleeping outside [12], poor socioeconomic status [13] and residence [14]. However, consistent findings are not yet attained because determinant factors vary with time, place and person in Ethiopia in general and the study area in particular. Taking into account the inconsistencies, we seek to assess determinant factors of childhood Plasmodium infection to enable exacting preventive and control measures against malaria among the school- age children in Northwest Ethiopia.

## Methods

### Study design, period and area

This cross sectional study was conducted among febrile school age children in Jawe district, Awe Zone, Amhara regional state, Northwest Ethiopia from April 2016–August 2016. The altitude and temperature of the district ranges from 648 to 1300 m above sea levels and 16.68^0^c - 37.6^0^c, respectively*.* According to health centers records, malaria remains the number one cause of admissions and child morbidity and mortality in the district. Malaria transmission occurs throughout the year with peaks after the two rainy seasons. Jawe district has many water bodies like Beles and Asihue rivers which possibly help for the breeding of malaria vectors throughout the year.

### Sample size and sampling technique

The systematic random sampling technique was employed to recruit the febrile school age children in this study. The data were collected from two health centers namely Jawe and Workmeda. The sample size in each health center was allocated by considering the number of population in the catchment areas. All febrile children aged 6–14 years, attending Jawe and Worekmeda health centers and willing to participate in the study were included in the study. Febrile children undertaking antimalarial or/and antibiotic drugs during the data collection time were excluded.

### Data collection

#### Questionnaire

A structured questionnaire was used to collect data on demographic information, determinant factors, explanatory variables and environmental related factors. The questionnaire were collected by trained health officers via face to face interview of parents/guardian of the children.

#### Parasitological examination

##### *Blood sample collection and examination*

Blood sample was collected by finger prick from each study participant for parasite detection. Both thick and thin blood films were prepared on a single slide. In the thick film preparation, three drops of blood were distributed over an area of 1cm^2^ at one end of the slide. Thin films were alsoblood prepared by evenly distributing a drop of blood on the other end the slide. Slides were labeled, dried, fixed with methanol alcohol (thin films only), and stained using 10% Giemsa stain solution for 30 min). The stained blood films were washed with distilled water and then air dried. The plasmodia were detected on the thick blood films, whereas species identification was done on the thin film.

### Quality assurance

To ensure reliability of data collection, training was given for data collectors. Application of standard procedures and accuracy of test results was supervised by the principal investigator. To eliminate observer bias, thick blood film slides were examined independently with two experienced laboratory technicians and 10% of the thick blood film slides was randomly selected and read by another technician as a quality control. The results of their observations were recorded for later comparison on separate sheets.

### Data analysis

Data were entered and analyzed using SPSS version 20 statistical software. The incidence of Plasmodium infection was computed using descriptive statistics and the chi-square. Strength of association between Plasmodium infection and determinant factors were computed by bivariate analysis. Those variables with a statistical significance and relevance to our research question in the bivariate logistic regression were taken to multiple regression analysis and the AOR was calculated to control potential confounders. *P*-values less than 0.05 were taken statistical significant.

## Results

A total of 333 febrile school-age children were included in this particular study. The range and mean age of the study participants’ were 8 and 11 years, respectively. The majority of cases (57.4%) were in the age range 11–14 years. Most of the participants (98.5%) were Orthodox Christianity followers. Two hundred seventy three (82%) febrile cases were rural inhabitants (Table 1).

**Table 1:** Socio-demographic variables of children infected with malaria in Jawe district, Northwest Ethiopia, 2016

Variables		N	Malaria status		*P* value
			Positive n (%)	Negative n (%)	
Age	6–8	70	33 (47.1)	37 (52.9)	0.30
	9–10	72	36 (50.0)	36 (50.0)	
	11–14	191	77 (40.3)	114 (59.7)	
Sex	Male	163	72 (44.2)	91 (55.8)	0.46
	Female	170	74 (43.5)	96 (56.5)	
Religion	Christian	328	144 (43.9)	184 (56.1)	0.62
	Muslim	5	2 (40.0)	3 (60.0)	
Residence	Rural	273	117 (42.9)	156 (57.1)	0.26
	Urban	60	29 (48.3)	31 (51.7)	
Total					

A total of 131 (91%) school children were infected with Plasmodia among school children who live in an area below 1 km from stagnant water. The number of plasmodium infected school children in DDT sprayed homes was 122 (87.8%). The prevalence of Plasmodium infection among-age school children of a household with a family size of five or more, those who regularly sleep after 22:00 h, sleep outside home and didn’t sleep under a bed net were 110 (81.5%), 119 (89.5%), 127 (84.7), and 126 (94.7). respectively (Table 2).

**Table 2:** The distribution Plasmodia with respect tocertain determinant factors in Jawe district, Northwest Ethiopia

Variables		Malaria status		
		Total (N, %)	Positive (N, %)	Negative (N, %)
Distance from stagnant water	<=1 km	144 (43.2)	131 (91)	13 (9)
	> 1 km	189 (56.8)	115 (60.8)	74 (39.2)
DDT sprays	No	139 (41.7)	122 (87.8)	17 (12.2)
	Yes	194 (58.3)	24 (12.4)	170 (87.6)
Family Size	≥ 5	135 (40.5)	110 (81.5)	25 (18.5)
	< 5	198 (59.5)	36 (18.2)	162 (81.8)
Bed-time	After 22:00	133 (39.9)	119 (89.5)	14 (10.5)
	Before 22:00	200 (60.1)	27 (13.5)	173 (86.5)
Sleeping outdoor	Yes	150 (45)	127 (84.7)	23 (15.3)
	No	183 (55)	19 (10.4)	164 (89.6)
Sleeping under a bed net	No	133 (39.9)	126 (94.7)	7 (5.3)
	Yes	200 (60.1)	20 (10)	180 (90)
Total		300 (100)	146 (43.8)	187 (56.2)

The odds of malaria infection was 98% lower in children who live in DDT sprayed homes than non-sprayed counter parts (AOR: 0.02 [95% CI: 0.0–0.88]. The odds of Plasmodium infection was about 40 times higher in children who live near a stagnant water (AOR: 43.2 [2.75–680.7]. The odds of malaria infection was 36 times higher in children who sleep outside their home (AOR: 36.6 [2.4–554.2] (Table 3).

**Table 3:** Determinants of malaria infection among children in Jawe district Northwest Ethiopia

Variables		Malaria status		COR [95%CI]	AOR [95%CI]	*P*-value
		Positive (n)	Negative (n)			
Distance from stagnant water	<=1 km	131	13	0.04 [0.0–0.33]	43.2 [2.8–680.7]	< 0.01
	> 1 km	115	74			
DDT sprays	No	122	17	0.02 [0.0–0 .19]	0.02 [0.0–0.88]	< 0.01
	Yes	24	170			
Family Size	≥ 5	110	25	0.07 [0.01–0.70]	14.74 [1.4–151.2]	0.02
	< 5	36	162			
Bed- time	After 22:00	119	14	0.03 [0.0–0.36]	31.9 [2.8–371.3]	< 0.01
	Before 22:00	27	173			
Sleeping outdoor	Yes	127	23	0.03 [0.0–0.42]	36.6 [2.4 – 554.2]	< 0.01
	No	19	164			
Sleeping under bed net the previous night	No	126	7	0.05 [0.01–0.35]	21.1 [2.8 – 154.7]	< 0.01
	Yes	20	180			

## Discussion

Malaria remains one of the most important causes of morbidity and mortality among school-age children. Currently, there is an increasing interest in malaria in school-age children as malaria may interfere with a child’s educational development [15]. Malaria is also one of the major causes of school absenteeism in school-age children of malaria-endemic areas. An earlier study conducted in Nigeria revealed that an average of three school days was lost per episode of malaria [16]. Another study also indicated that infected primary school children had lower test scores for abstract reasoning and sustained attention compared with uninfected children in Uganda [17].

Multivariate logistic regression revealed significant associations between malaria infection and several independent variables. For instance, regular bed net utilization was associated with lower odds of malaria infection in our study. The reported prevalence of sleeping under a net in this study was 60.1%, indicating significant numbers of children were not protected in the study area. This goes in line with surveys in most malaria endemic areas of Africa where school-age children sleep under nets less often than any other age group. For instance, results obtained from Malawi (16), and Cameron (17) revealed that school-age children slept under a bed net less frequently. In these endemic regions, there is a tendency for mothers slept under nets with their young children, while other household members slept under nets less frequently. Additionally, older children and adults are perceived to be less at risk and may use bed nets less frequently [8]. This may, in part explain higher infection prevalence of malaria among school-age children in the current study area.

The odds of Plasmodium infection was 21 times higher among children who did not sleep under long-lasting insecticide treated net (LLIN) than those who utilized it regularly. This was supported by previous studies where there existed evidence that regular use of LLIN significantly lowered the risk of malaria at the individual level [15, 18, 19]. However, universal coverage is challenged by the fact that school-age children have attracted relatively little attention as a group in need of special measures to protect them against malaria so far. As children become older and more independent, parents have less control over the time when they go to bed, where they sleep, and whether they use a net resulting in lower net coverage in children in this age group. For instance, surveys conducted in several African countries revealed that school-aged children were the group least likely to sleep under an ITN, which left more than 30% of school-aged children unprotected [9]. Similarly, low ITN usage rates have been observed among school-age children in Cameroon [20], Kenya [21], Malawi and Uganda [17, 22].

Our study also showed that indoor residual spraying (IRS) significantly decreased incidence of malaria among school children. The odds of malaria infection were 0.02 times lower among children from households with indoor residual spraying. This was supported by a study in Tanzania, where repeated IRS campaigns reduced malaria parasitaemia from 73 to 4% in school-age children [23]. Another recent study in Kenya also showed that targeted IRS halved malaria incidence in the school children [24].

Despite the fact that Anopheles mosquitoes can fly considerable distances, multivariate analysis showed a statistically significant association between the estimated distance of residents to stagnant water and Plasmodia infection in the current study area. This was consistent with a previous conducted study [25].

Family size had also showed statistically significant association with malaria blood slide positivity which is similar with other studies in Ethiopia [26, 27]. This might be due to inadequate number of bed nets to family size leaving certain members of the household unprotected. As a result, school-age children who didn’t sleep under bed net due to in adequate bed net distribution may acquire Plasmodium infection.

In our study, about half of the participants reported outdoor sleeping at some time and the multivariate analysis indicated causal link between sleeping in the outdoors and Plasmodium infection. The odds of Plasmodium infection was 37 times higher among those who experienced outdoor sleeping at any time. This could predispose children to the infective mosquito bites in areas where the anophelines exhibit exophagic and exophilic behaviors. This outdoor sleeping could further jeopardize the wellbeing of children with respect to malaria, because bed nets are not usually hanged during outdoor sleeping [12]. Specific barriers of using bed nets in the outdoors such as not wanting or not knowing how to hang, the fluidity of sleeping spaces and movement from outdoors to indoors during the night were reported elsewhere [12].

Our study also indicated significant associations of late night sleeping and Plasmodium infection. While the children sleep late, the chance of vector-human contacts are enhanced and late nights usually coincide with the peak biting hours of nocturnal anophelines [12].

## Conclusion

The incidence of Plasmodium infection was higher among febrile children in Jawe district. Distance from stagnant water, family size, outdoor sleeping, bed net use, late night sleeping and DDT spraying are the major determinant factors for the high incidence. Therefore, community mobilization and health education should be promoted on proper bed net utilization, early diagnosis and timely treatment.

## Abbreviations

AOR: Adjusted Odds Ratio
CI: Confidence Interval
DDT: Dchlorodiphenyltrichloroethane
LLIN: Long lasting insecticide treated net
MOH: Ministry of Health
OR: Odds ratio
